# New Seeding Approach Reduces Costs and Time to Outplant Sexually Propagated Corals for Reef Restoration

**DOI:** 10.1038/s41598-017-17555-z

**Published:** 2017-12-22

**Authors:** Valérie F. Chamberland, Dirk Petersen, James R. Guest, Udo Petersen, Mike Brittsan, Mark J. A. Vermeij

**Affiliations:** 1 SECORE International, Hilliard, OH USA; 2 CARMABI Foundation, Piscaderabaai, Willemstad, Curaçao; 3 Freshwater and Marine Ecology, Institute for Biodiversity and Ecosystem Dynamics, University of Amsterdam, Amsterdam, The Netherlands; 4 School of Natural and Environmental Sciences, Newcastle University, Newcastle upon Tyne, United Kingdom; 5 Faculty of Mechanical Engineering, Kempten University of Applied Sciences, Kempten, Germany; 6 Columbus Zoo and Aquarium, Powell, OH USA

## Abstract

The use of sexually propagated corals is gaining popularity as an approach for reef restoration. However, manually attaching substrates with recently settled corals to the reef using binding materials is both time-consuming and expensive, limiting the use of this technique to small spatial scales. We present a novel approach whereby young corals are ‘seeded’ on the reef without the need for manual attachment to the benthos. We tested two tetrapod-shaped concrete substrates (7.9 and 9.8 cm in diameter) on which coral larvae were settled. The tetrapods were efficiently deployed by wedging them in reef crevices, in 1.5 to 7% of the time required for traditional outplanting techniques. Seeding tetrapods was most effective in reefs with moderately to highly complex topographies, where they rapidly became lodged in crevices or cemented to the benthos by encrusting organisms. After one year, average recruit survival was 9.6% and 67% of tetrapods still harboured at least one coral colony, and overall, this approach resulted in a 5 to 18 fold reduction in outplanting costs compared to common outplanting methods. This seeding approach represents a substantial reduction in costs and time required to introduce sexually propagated corals to reefs, and could possibly enable larger scale reef restoration.

## Introduction

The loss of ecological functions and ecosystem services provided by coral reefs worldwide has prompted conservation and management efforts to promote their recovery by addressing local causes of decline^1^. These measures can be ‘passive’ whereby natural recovery is facilitated through human intervention (e.g., implementation of fishing quotas, pollution regulation)^2^, or take the form of ‘active’ measures whereby humans directly manipulate the dynamics of degraded reef ecosystems (e.g., coral propagation, artificial reefs, removal of invasive species)^3^. Because many coral reefs are assumed to no longer recover naturally from anthropogenic stressors^1^, active restoration approaches are increasingly considered, in conjunction with passive management interventions, to rehabilitate degraded reef communities.

Outplanting corals into degraded areas is a common active restoration approach aimed at increasing coral cover and structural complexity^4^. Corals for outplanting are typically clonal asexual fragments or naturally dislodged “fragments of opportunity” of extant colonies^5^. Fragments are often grown-out in coral nurseries prior to outplanting and, when outplanted, have been observed to locally increase the abundance and diversity of fish^6^. However, the use of clonally produced fragments also results in limited genetic diversity within recipient populations, and thus may reduce their potential to adapt to changing environmental conditions^7^. In contrast, the use of sexually produced corals, whereby genetic recombination ensures the formation of new genetic varieties, preserves genetic variation within outplanted corals during restoration efforts^4^. Consequently, the use of sexually produced corals can complement more commonly used clonal approaches and provide the possibility for genetic adaptation to climate change^7^.

Following gamete collection and *ex situ* fertilization, sexually produced coral larvae are generally settled on artificial settlement substrates (“settlement tiles”)^8^, that are either directly outplanted to the reef ^9,10^, or kept in land- or ocean-based nurseries^11–13^ where coral settlers are grown to sizes (generally >1 cm^2^) that make them less vulnerable to predation and competition^14,15^. The success of sexual coral propagation techniques has improved over recent years. While large numbers of outplanted corals regularly survive past the age of one year^9,11,16,17^ and outplanted corals have reached sexual maturity in a few occasions^12,18,19^, mortality among newly settled corals remains extremely high (i.e., type III survivorship)^20^ compared to restoration approaches using clonal fragments. Typically less than 5% of all cultured settlers survive for more than one year^4^, and high (natural) levels of post-settlement mortality therefore greatly reduce the effectiveness of restoration methods using sexually produced larvae.

The high costs of both asexual and sexual restoration approaches limit their application to spatial scales (<1 hectare) that are generally too small to re-establish ecological functions of degraded reef systems^21,22^. The process of outplanting artificial substrates with settled corals to the reef typically accounts for 30% of the total restoration costs when individual corals or substrates are manually secured using binding materials (e.g., cable-ties, epoxy, nails). In contrast, gamete collection, larval rearing and larval settlement combined typically account for less than 50% of costs^4^. Current outplanting techniques require tedious handling of binding materials underwater and are therefore time consuming. For example, previous studies found that between 4 and 20 min are needed to outplant a single substrate with coral settlers to the reef ^9,11,13^. Restoration efforts using sexually propagated corals would especially benefit from new technologies that enable cheap and fast outplanting and increased settler survival.

**Figure 1:**
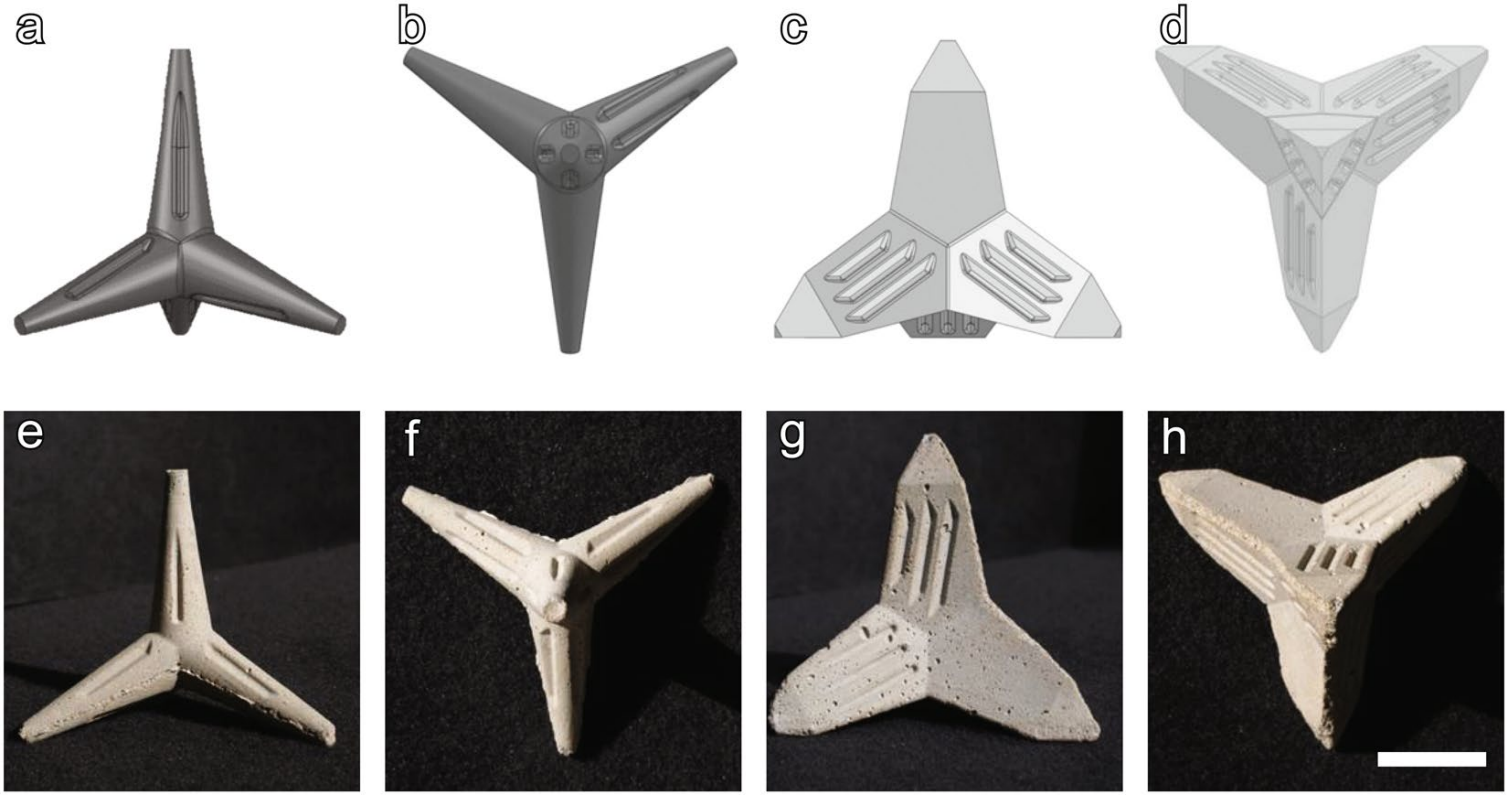
Tetrapod-shaped substrates for coral larval settlement. Computer-aided-designs (CADs) for Type I: (**a**) side view and (**b**) top view, and for Type II: (**c**) side view and (**d**) top view. Tetrapods before they were conditioned in a flow-through aquarium system: Type I: (**e**) Side view, and (**f**) top view, Type II: (**g**) side view, and (**h**) top view. Scale bar = 3 cm. CADs by Kempten University of Applied Sciences and photos by DP.

In this study we tested the efficiency of outplanting three-week-old coral settlers using novel tetrapod-shaped substrates for coral settlement (Fig. 1) that can be outplanted by simply wedging them into natural crevices, without the need for binding materials. Tetrahedral shapes are commonly used in coastal defences to dissipate water movement and wave energy. Their “spikey” shape makes them relatively stable substrates once placed on the benthos^23^. Two different tetrapod-shaped substrates were designed: Type I (Fig. 1a,b,e,f) with thin conical-shaped pods, and Type II (Fig. 1c,d,g,h) with thicker triangular-shaped pods. Thinner and pointier conical pods were assumed to enhance the probability of the tetrapods to become attached or stuck to the reef. Thinner pods might also have, however, poorer structural strength, causing them to be more vulnerable to breakage. We therefore tested the two designs to quantify potential trade-offs between thicker (less breakage) and thinner (faster attachment) pods.

We hypothesized that the success of aforementioned ‘seeding’ approach would depend on the structural complexity of the habitat in which tetrapods were introduced. On shallow coastal reefs the attenuation of wave energy is largest on structurally complex landscapes^24,25^. Complex reef topographies also contain a larger number of crevices, fissures and holes in which tetrapods can be wedged^26^. We therefore expected that a larger proportion of tetrapods would be retained in highly complex topographies than on reefs with low or sparse relief. To test this hypothesis we assessed if the movement of the tetrapod-shaped substrates, even if not secured with binding materials, would be low enough that they would become rapidly attached or stabilized within the reef framework in areas with low to high levels of structural complexity. Settler survival and growth were followed for one year after outplanting. Lastly, we compared the cost-effectiveness of this new approach relative to existing outplanting methods.

## Materials and Methods

### Design and production of tetrapod-shaped substrates for coral settlement

Both tetrapod types consisted of four pods positioned in tetrahedron angles (109.47°) relative to each other (Fig. 1). Tetrapod Type I (Fig. 1a,b,e,f) had thin conical-shaped pods, whereas tetrapod Type II (Fig. 1c,d,g,h) had thicker triangular-shaped pods. The tips of the pods of both tetrapod types narrowed toward their ends to increase the probability that they would get stuck in crevices and thus increase overall attachment success. Because the availability of microhabitats on artificial substrates promotes larval settlement^8^ and post-settlement survival^27,28^, grooves were incorporated on each of the four pods of both designs (Fig. 1) (Type I: 3 grooves per pod, 27.5 × 2.4 × 1.3 mm, Type II: 6 grooves per pod, 28.7 × 2.4 × 1.6 mm, L × W × D, see Supplementary Table S1). Tetrapods needed to be large enough to reduce their chance of falling into deeper reef crevices unsuitable for coral growth, but small enough that they could be easily handled during the rearing phase where larvae are settled on the tetrapods and during the outplanting itself. Tetrapod Type I was slightly smaller and lighter than tetrapod Type II (Type I: Ø 7.9 cm, 51.1 g, Type II: Ø 9.8 cm, 85.6 g, Table S1). Because coral settlers on each settlement substrate can, in theory, grow into a single coral colony after successful outplanting, the use of smaller-sized substrates harbouring small numbers of settlers is more effective for restoration efforts than fewer, larger-sized substrates harbouring numerous settlers^8,29^. Smaller-sized substrates furthermore allow young corals to rapidly overgrow the artificial substrate and attach to underlying reef substratum which increases their probability of recruiting to the adult population^9^.

The tetrapods were designed using three-dimensional Computer-Aided Design software (3D-CAD; SolidWorks, Massachusetts, USA), and made of concrete. Moulds were made from polyurethane and manufactured with a multi-axis-milling machine (VTC 800/30 SR, Mazak, Germany). The tetrapods were casted in concrete between March and July 2013 and made from a homogenous mixture of 2 parts Portland cement, 4 parts river sand and 1 part water. This mixture could be easily poured in the moulds and dried rapidly. Biodegradable vegetable oil was sprayed into the moulds prior to pouring to prevent the concrete from sticking to the moulds’ sides. The concrete was allowed to dry for 24 h before the tetrapods were extracted from the moulds.

### Rearing and settlement of coral larvae

Experiments were conducted on Curaçao (12°N, 69°W), a Caribbean island located 60 km north off Venezuela. The tetrapods (n = 80 of each type) were incubated in a flow-through seawater aquarium system for six months to wash out potentially toxic and alkaline agents from the cement mixture and allow the development of biofilms that induce coral settlement and metamorphosis^30^. The aquarium system consisted of five flow-through aquaria (acrylic, 215 × 69 × 64 cm, L × W × H) that were continuously supplied with natural seawater (~2300 L h^−1^) from a nearby reef. See Chamberland *et al*.^9^ for a detailed description of this system.

*Favia fragum* (Esper 1797) releases planula larvae 6 to16 days after the new moon throughout the year^31^. Fifty adult *F. fragum* colonies were collected from the Curaçao Sea Aquarium reef (12°4′59″N, 68°53′44″W) two days before the onset of their planulation cycle in March 2014 and kept in the aforementioned flow-through system. Between days 6 and 10 after new moon and one hour before sunset, colonies were placed overnight in two 70-L plastic cool boxes (Princeware Glacier, UK) containing ~60 L of 50-µm-filtered seawater. Every morning (between 7:00 and 8:00), all larvae released during the preceding night were collected using glass pipettes and distributed randomly among eight plastic containers (36 × 31 × 24 cm, L × W × H, Sterilite) filled with ~23 L of 50-µm-filtered seawater, larvae collected during previous nights, and 10 Type I and 10 Type II tetrapods. The parent colonies were then removed from the cool boxes and returned to the flow-through system. All collected larvae were divided among the eight containers resulting in a total of ~600 coral larvae per container. Containers with coral larvae were partially submerged in the flow-through system to maintain natural seawater temperatures (28–29 °C) and water inside the containers was exchanged daily (~75%) to maintain water quality. Two airlifts were placed in the opposite corners of each container to generate water movement and prevent the formation of stagnant water in between the tetrapods. Larvae were left in the containers for five days to settle after which all tetrapods were transferred to the flow-through system.

Larval settlement rates on each tetrapod were assessed immediately before outplanting using a blue light (Night Sea, MA, USA) that causes settled larvae to fluoresce. To determine if settlement preferences differed between the two tetrapod designs (Type I, Type II), the different surface orientations (Topside, Underside) and the microhabitats types (Grooved, Flat), the position of each settler on each tetrapod was mapped. Settlement rates were calculated as the number of settlers per cm^2^ of available surface area per tetrapod type, surface orientation and microhabitat type. Until they were seeded to the reef, all tetrapods with ≥1 live coral settler (i.e., henceforth referred to as ‘seeding units’, SUs) were hung ~50 cm below the water surface using 27.2-kg strength fishing line tied to PVC frames placed on top of the flow-through aquaria.

### Seeding of SUs on the reef

**Figure 2:**
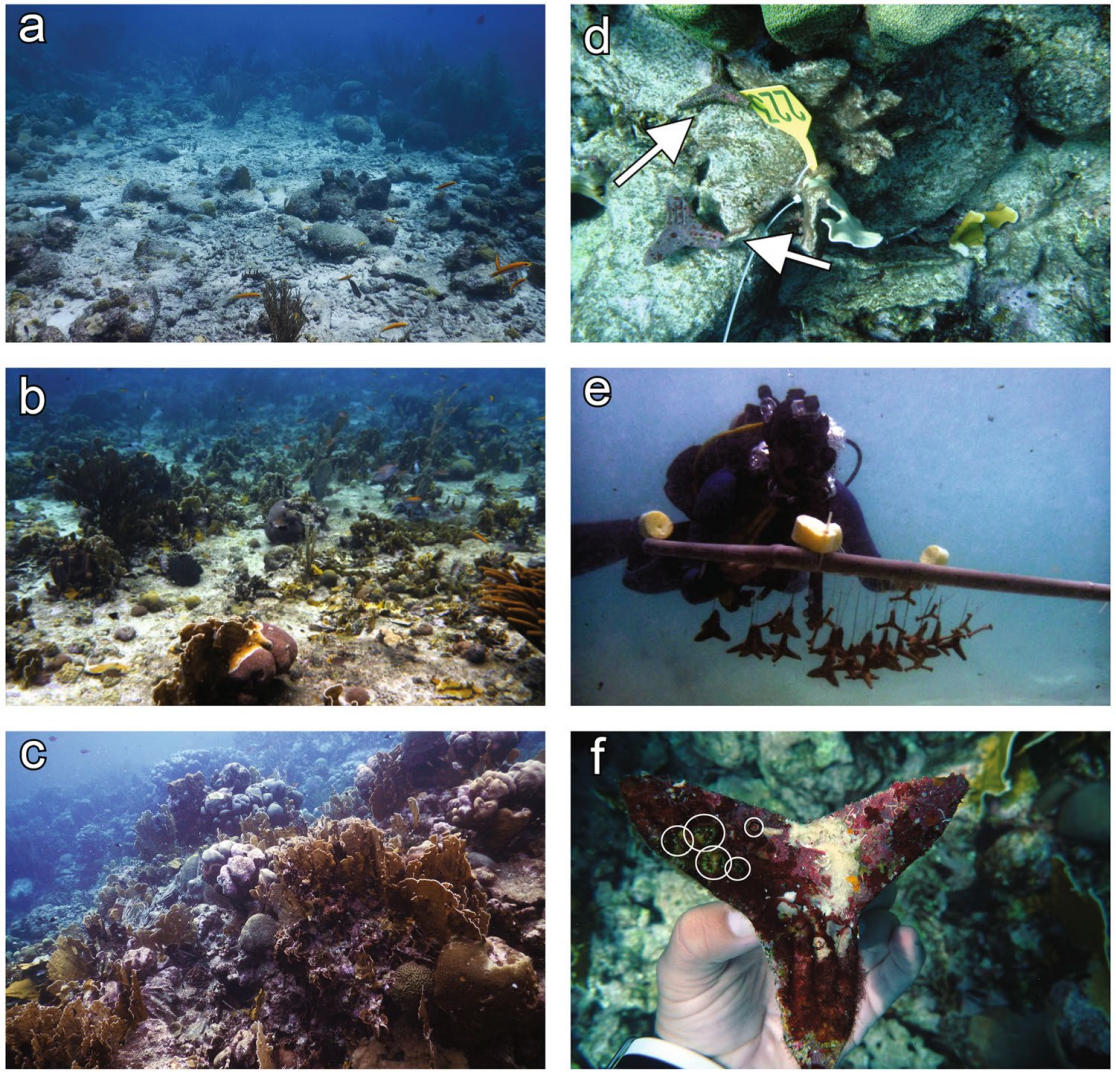
Seeding and monitoring of seeding units (SUs) in different levels of structural complexity. The two tetrapod types with coral settlers were seeded in reef areas with (**a**) Low, (**b**) Medium, and (**c**) High levels of structural complexity. (**d**) Outplant locations were marked with numbered plastic tags and SUs were (**e**) transported to the reef on 1 × 1 m PVC frames by a SCUBA diver and (**d**) wedged into crevices in the reef framework less than 30 cm away from their respective tag (tetrapods are shown by the white arrows). (**f**) At each survey, a picture of each of the tetrapod’s four sides was taken to assess settler (shown by white circles) survival and growth. Photos by VFC.

Three weeks after *F. fragum* larvae had settled, SUs were seeded at the Curaçao Sea Aquarium reef, a relatively healthy reef approximately 100 meters from our rearing facility. Tetrapods were seeded within a 150 × 10 m area parallel to the coast at depths between 4 and 6 m and individually placed in a habitat of Low, Medium, or High structural complexity. These different habitat types occurred interspersed as small patches (2–10 m in width) within the outplanting area. Assignments to structural categories were made visually following Wilson *et al*.^26^. Low, Medium, or High structural complexity corresponded, respectively, to low and sparse relief, moderately complex, and very complex with numerous fissures and caves (Fig. 2a–c). To facilitate the search for tetrapods at each survey, individual outplant locations where one SU of each type was seeded were marked with numbered plastic tags that were fixed to the reef with cable ties (Fig. 2d). Tetrapods were outplanted at least 3 m from one another to avoid their potential misidentification due to tetrapod dispersal during the course of the experiment.

Ten SUs of each tetrapod type were seeded at the three levels of structural complexity. Only SUs with similar overall settler densities were used (Type I: 0.29 (±0.11 SD), Type II: 0.24 (±0.08 SD), mean number of settlers per cm^2^) to minimize potential confounding effects of density dependent processes^29^. SUs were transported from the aquaria to the reef while hanging from the same PVC frames (1 × 1 m; Fig. 2e) used during the initial rearing phase. Tetrapods were then seeded by a diver who cut each SU from the PVC frame and wedged them into crevices of the reef framework. Large (>10 cm Ø) and deep ( > 30 cm depth) crevices were avoided to reduce the chance of tetrapods being lost into the reef framework. One SU of each tetrapod type was seeded in close proximity (≤30 cm) to each tag (Fig. 2d), after which an overview-photograph (Lumix DMC-TS2, Panasonic) of the area (~1.5 × 2 m) was taken in planar view (see Supplementary Fig. S1) to document the surrounding benthos and the location of each tetrapod relative to each tag.

### Monitoring of tetrapod dispersal and coral settler survival and growth

Tetrapod dispersal was monitored 1.5 week, 3 and 6 months after outplanting. Settlers’ survival and growth rates were monitored after 3, 6 and 12 months. At each time point, the area around each tag was carefully searched for the SUs. If a SU was not found within 3 m of a tag, it was considered lost and excluded from the survivorship analysis. To calculate the dispersal of each SU through time, an overview-photograph of each outplant location was taken in planar view (~1.5 × 2.0 m) for each time point so the position of each SU could be tracked through time using natural landmarks and the tags for scale (Supplementary Fig. S1). The distance between the positions of the tetrapods through time was determined using ImageJ^32^ (Fig. S1). During each survey, by gently trying to move each tetrapod, we assessed whether it had become “attached” (i.e. stuck in or cemented to the reef framework) or whether it was laying loose on the reef substratum (i.e., “non-attached”). Each tetrapod was then detached from the reef and a high resolution photograph was taken of each of its four sides (Lumix DMC-TS2, Panasonic; Fig. 2f) after which it was carefully returned to its original position on the reef. This may have caused the detachment of some of the already attached tetrapods, but lifting each tetrapod was necessary as surviving settlers were often found on their undersides.

To quantify survival rates of coral settlers on each tetrapod type in the three levels of topographic complexity, the number of live *F. fragum* on each tetrapod was assessed on the photographs during each time point and compared to the map overviewing the distribution of initial settlers. Settler size (in surface area in mm^2^ and number of polyps) was also quantified from photographs for each time point using ImageJ. Lastly, we calculated the proportion of outplanted SUs that could be found and still harboured ≥1 settler (i.e., still represented a SU) through time for all treatments, henceforth referred to as ‘SU yield’. SU yield serves as a measure of success to compare the effectiveness of different restoration approaches, assuming that a single, large, coral colony can theoretically grow to adulthood per outplanted SU^4^.

One week before the last survey (t = 12 months), a storm caused major breakage of *Millepora* spp. and *Acropora palmata* colonies within the study area. A total of nine tags (out of 30) could no longer be located and were likely buried under scattered *Millepora* and *Acropora* fragments or had detached. Tetrapods associated with these tags were excluded from the analysis at this time point. Dispersal distances for all tetrapods could not be measured, because most natural landmarks had also been covered or were no longer present.

### Cost-effectiveness of seeding sexually propagated corals

The costs of seeding the two tetrapod types was calculated following Edwards^4^, and compared to the few existing studies that quantified costs associated to outplanting techniques for sexually propagated corals that have used binding materials. The latter studies included restoration approaches that (1) tied other types of substrates to a rope previously nailed on the reef ^9^, (2) epoxied substrates to the reef ^19^, and (3) secured substrates in holes previously drilled in the reef framework^11,13^. Expenses associated with larval rearing (e.g., gamete/larvae collection, culture maintenance, larval settlement, nursery construction and maintenance) can significantly vary depending on species, rearing techniques, nursery types, and the duration of the nursery period^4,9,11,13,17^. This study specifically aimed at increasing the cost-effectiveness of the outplanting phase. Thus, in order to compare outplanting costs among studies, expenses related to the larval rearing phase were not considered in the cost-analysis. The analysis therefore only included expenses related to (1) the production or purchasing of settlement substrates, (2) materials needed to secure the substrates to the reef (e.g., cable-ties, nails, pneumatic drills, epoxy), (3) air tanks for SCUBA divers, and, if needed, pneumatic drills, and lastly, (4) labour required to carry out the outplant (Supplementary Table S2). Reusable items such as SCUBA and snorkelling gear were assumed to have a three year life span so their cost was divided by three to calculate their costs for one outplanting effort per year^4^. To standardize between studies, pneumatic drills were assumed to consume one air tank per dive, and we used a ratio of one diver handling a drill per team of three divers. We did not include costs related to boat usage as this expenditure is highly dependent on local conditions such as fuel prices and distance to the restoration site.

Labour was expressed in terms of person-hour and converted to US dollars based on the median worldwide GDP at the time the work was carried out^33^ (i.e., $6.63 h^−1^), and only included the time required to carry out the outplant itself. The time to prepare for dives and reach outplanting sites were not considered as the latter significantly vary among locations. The time needed for divers to wedge one SU in the reef framework was calculated from video footage taken during outplanting and was measured as the time from when a diver first held a SU in his hand ready to seed it, until the SU was wedged in the reef. The time required to outplant substrates using other outplanting techniques than seeding was taken from above-mentioned studies. To compare the total costs of the different restoration approaches, the costs to restore one hectare of reef with 10,000 SUs with 10 persons was calculated for each method, and its effectiveness expressed as SU yield after one year. Because settler mortality is highest during the first year of outplanting^14^, the SU yield after one year was assumed to be an adequate metric to evaluate the long-term success of sexual coral restoration efforts.

### Data analysis

To compare settlement preferences between the tetrapod designs (Type I, Type II), surface orientations (Topside, Underside) and microhabitats (Grooved, Flat), Welch’s F-test for unequal variances^34^ was used followed by Tukey’s post-hoc HSD tests because data did not meet the assumption of homoscedasticity. Independent replicates (i.e., tetrapods) per surface orientation/microhabitat type were used for the analysis (n = 30 and 32 tetrapod Type I and II, respectively). Differences in settler survival among tetrapod types, microhabitats and levels of structural complexity were compared with Kaplan-Meier’s survival analysis^35^ followed by log-rank (Mantel-Cox) pairwise comparisons. Fisher’s exact test of independence was used to test for differences in attachment rates of the tetrapods on the reef, as well as differences in the proportion of tetrapods still harbouring at least one coral individual through time (i.e., SU yield). One-way ANOVAs were used to assess potential differences in settler growth, whereas differences in dispersal rates were tested with repeated measures ANOVAs. All analyses were performed in SPSS 24.0^36^. Statistical values for ANOVAs and post-hoc pairwise comparisons are available as online supplementary information (Supplementary Tables S3–S10). All data generated and analysed during this study are included as a supplementary information file.

### Ethics statement

All research was carried out under the research and collecting permits granted to the CARMABI Foundation by the Government of Curaçao.

## Results

### Settlement preferences of *F. fragum* larvae

An average of 70% (±6SE, n = 8 settlement containers) of *F. fragum* larvae settled on either tetrapod design. This resulted in 60 Type I and 64 Type II SUs that, immediately after larvae settled, harboured from 5 to 48 and 8 to 63 settlers with an average of 21.2 (±1.2SE) and 28.0 (±1.7SE) settlers, respectively. *F. fragum* larvae settled in slightly higher densities (number of settlers per cm^2^) on Type I than on Type II tetrapods (Welch’s F-test: F_1,116_ = 18.36, p < 0.001). Larvae settled foremost on the undersides of tetrapod Type II (Welch’s F-test: F_1,49_ = 11.7, p = 0.001), but did not discriminate between surface orientations on Type I tetrapods (Welch’s F-test: F_1,49_ = 0.38, p = 0.54). For both tetrapod types, settlement rates inside grooves were 2.4 (Type I) and 2.9 (Type II) times higher than on flat surfaces (Welch’s F-test: Type I, F_1,33_ = 22.1, p < 0.001, Type II, F_1,38_ = 31.7, p < 0.001).

### Tetrapod dispersal and attachment rates on the reef

**Figure 3:**
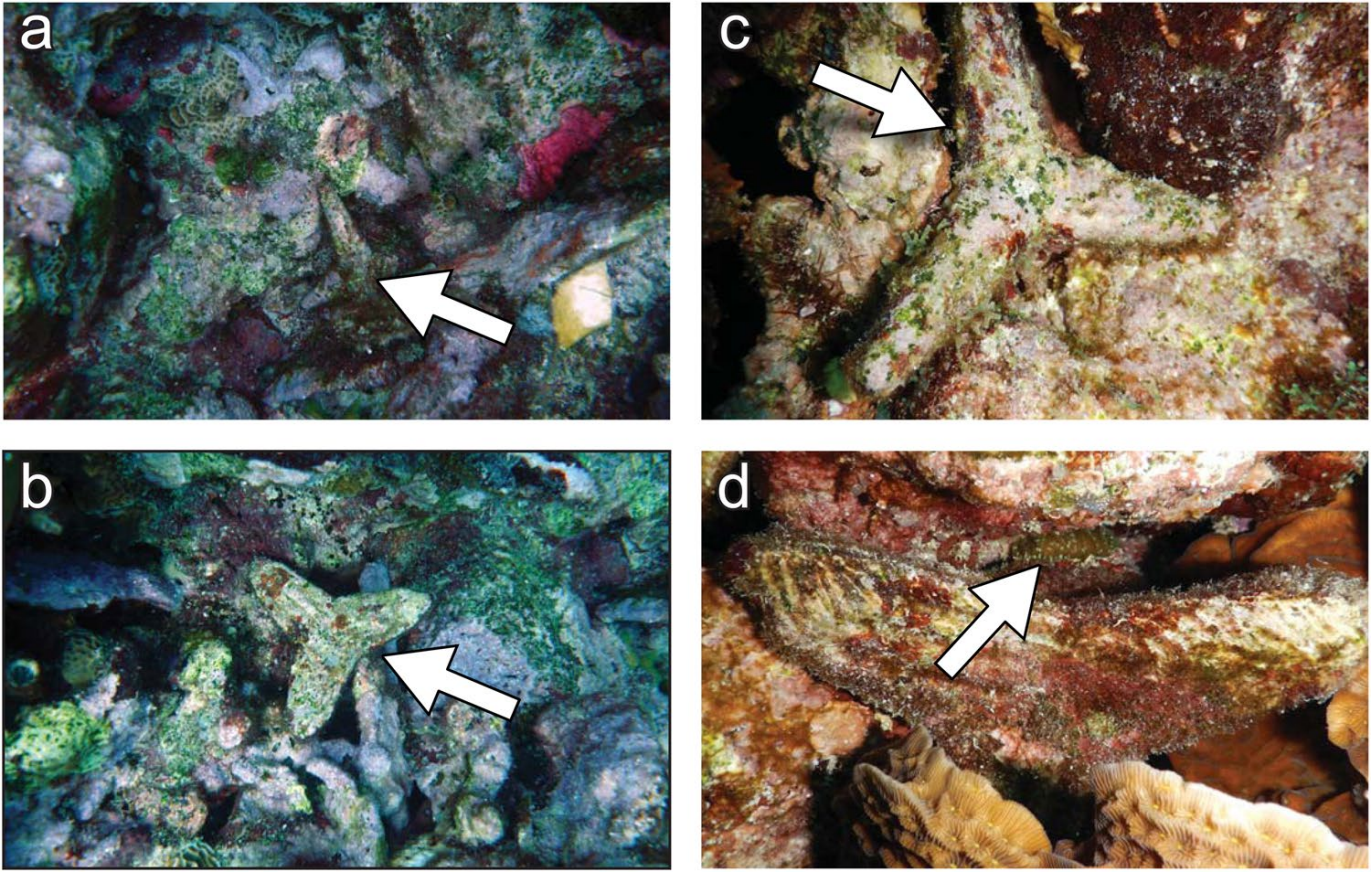
Example of the two tetrapod designs six months after they were seeded to the reef. After 6 months, 75% of the Type I (**a**) and Type II (**b**) tetrapods were firmly lodged in crevices and/or had become cemented to the reef framework by encrusting benthic organisms such as sponges, crustose coralline algae and hydrocorals, and were hardly distinguishable from the reef framework. Arrows show the tetrapods. (**c**,**d**) are close-up pictures of six-month-old *Favia fragum* colonies (indicated by arrows) growing on both tetrapod designs. Photos by VFC.

Tetrapods dispersed most during the first two weeks after outplanting with an average of 6.0 cm per week (±1.5SE, n = 57), after which they moved less than 2.0 cm per week (Table 1) (one-way RM ANOVA: F_1_ = 8.6, p = 0.006, Supplementary Table S3). After the first two weeks and during the subsequent 5.5 months, 50% of the tetrapods never moved. After one year 76% of the tetrapods could be recovered of which 84% were either firmly lodged in crevices or cemented to the reef framework by encrusting benthic organisms (Fig. 3a–d, Table 2).

**Table 1:** Mean dispersal of the two tetrapod designs seeded in three levels of reef structural complexity.

Tetrapod type	Net dispersal rate (cm week^−1^)									Total dispersal (cm)		
	0 to 2 weeks			2 to 12 weeks			12 to 24 weeks			24 weeks		
	$\bar{{\boldsymbol{X}}}$	SE	n	$\bar{{\boldsymbol{X}}}$	SE	n	$\bar{{\boldsymbol{X}}}$	SE	n	$\bar{{\boldsymbol{X}}}$	SE	n
Type I	6.3	2.5	29	1.3	0.5	19	0.6	0.2	25	32.4	8.9	25
Type II	5.6	1.5	28	1.5	0.6	21	0.3	0.1	23	32.2	8.9	23
**Structural complexity**												
Low	12.1	3.9	18	2.2	0.8	14	0.8	0.2	16	60.3	14.0	16
Medium	3.6	1.4	20	2.1	0.8	12	0.3	0.2	15	30.0	8.9	15
High	2.7	1.1	19	0.1	0.1	14	0.2	0.1	17	8.1	2.8	17
**Overall**	6.0	1.5	57	1.4	0.4	40	0.4	0.1	48	32.3	6	48

**Table 2:** Status of the two tetrapod designs seeded in three levels of reef structural complexity through time.

Tetrapod type	Recovery rate (% of outplant locations)								Attachment rate (% of recovered tetrapods)							
	2 weeks		3 months		6 months		1 year		2 weeks		3 months		6 months		1 year	
	*%*	n	*%*	n	*%*	n	*%*	n	*%*	n	*%*	n	*%*	n	*%*	n
Type I	100	29	79	24	83	30	81	21	90	29	84	19	76	25	94	17
Type II	96	28	87	23	83	29	70	20	70	27	80	20	78	23	71	14
**Structural complexity**																
Low	100	17	74	19	84	19	64	11	47	17	57	14	63	16	57	7
Medium	100	20	86	14	80	20	67	12	90	20	92	12	67	15	75	8
High	95	20	93	14	85	20	89	18	100	19	100	13	100	17	100	16
**Overall**	98	57	83	47	83	59	76	41	80	56	82	39	77	48	84	31

Despite their different shapes, there were no differences in the distance that tetrapod Type I and II dispersed during the first six months of the experiment (Table 1) (two-way RM ANOVA: F_1_ = 0.07, p = 0.79, Supplementary Table S4). After 1 year, the relocation success of both tetrapod Type I and II was similar across all three levels of reef complexity (Type I, 81%, lower 95% confidence limit (LCL) = 60%, upper 95% confidence limit (UCL) = 92%, Type II, 70%, LCL = 46%, UCL = 88%, respectively (Table 2) (Fisher’s exact test: p = 0.33). The two tetrapod designs were also equally likely to become stabilized within the reef framework, and after one year 94% (Type I, LCL = 73%, UCL = 99%) and 71% (Type II, LCL = 45%, UCL = 88%) of tetrapods were attached to the reef (Table 2, Fig. 3a–d) (Fisher’s exact test, p = 0.11). While the two designs proved equally effective in promoting the stabilization of the tetrapods on the reef, it is worth noting that the thinner pods of Type I were more fragile, causing them to break often during production (~10%, VFC pers. obs.) and while being handled in the field (~10%, VFC pers. obs.).

All tetrapods that could be recovered within high complexity habitats were attached to the reef after six months. Attachment success was lower (63%, LCL = 35%, UCL = 85%) in low complexity habitats (Table 2) (Fisher’s exact test: p < 0.01). After one year, recovery rates for tetrapods placed in low complexity habitats were lower (64%, LCL = 31%, UCL = 89%) compared to high complexity habitats (89%, LCL = 65, UCL = 99%) (Table 2). Tetrapods in low complexity habitats dispersed 3.4 and 4.7 times farther than in Medium and Highly complex habitats respectively during the first two weeks following the outplant (Tukey’s HSD test: p = 0.002, Supplementary Table S5), resulting in a total dispersal distance averaging 60 cm (±14SE) after six months compared to 8 cm (±3SE), respectively (Table 1).

### Survival and growth of coral settlers

**Figure 4:**
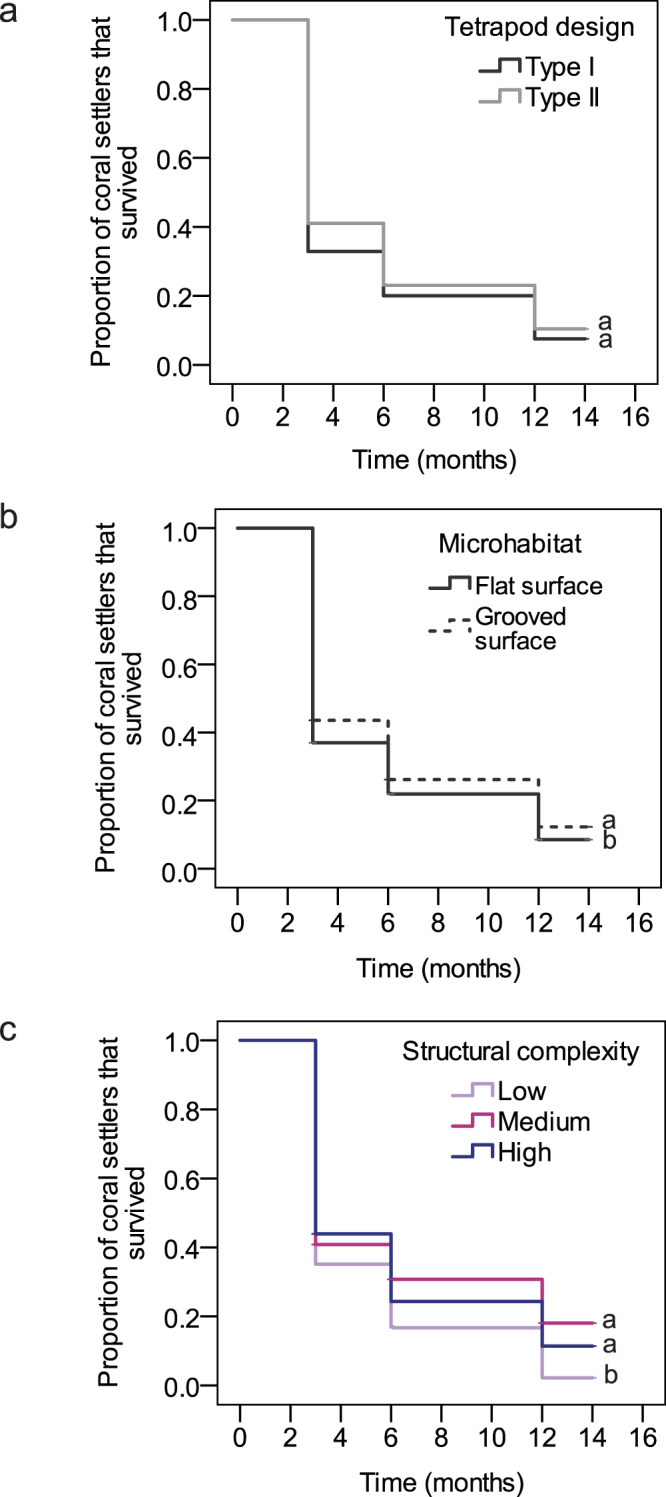
Survival of coral settlers. Proportion of initial *Favia fragum* settlers that survived through time (**a**) on Type I and II tetrapods, (**b**) inside grooves and on flat surfaces, and (**c**) that were seeded in Low, Medium and High levels of habitat complexity. Different letters next to bars indicate statistically different groups (p < 0.05) as determined with a Kaplan-Meier analysis followed by pairwise log-rank (Mantel-Cox) comparisons.

After one year, an average of 9.6% of initial *F. fragum* settlers (n = 30 substrates) had survived and grown to an average size of 30.2 mm^2^ (±2.8SE, n = 60 settlers). At that point, 62% of live individuals had completed at least one polyp division and consisted of 2 to 7 polyps. The average settler survival (Fig. 4a) on Type II tetrapods was similar (9.8%, n = 14 substrates) to that on Type I tetrapods (9.4%, n = 11 substrates) (K-M: χ^2^_1_ = 0.00, p = 0.99). Growth was also equal between the two designs (one-Way ANOVA: 6 months, F_1,188_ = 0.006, p = 0.94, 12 months, F_1,58_ = 0.02, p = 0.89, Supplementary Table S6). On both tetrapod Type I and II, and across all levels of structural complexity, larvae that had settled inside grooves showed a 1.8 fold higher survival rate after one year compared to those that settled on flat surfaces (Fig. 4b) (K-M: χ^2^_1_ = 7.4, p = 0.007), suggesting that the grooves served as sheltered microhabitats for newly settled corals. While survival rates of coral settlers on tetrapod Type I were unaffected by the distance that SUs had moved during the study period, 21.3% and 26.6% of the variation in settler survival rates on Type II tetrapods could be linked to the latter’s total dispersal after respectively 3 and 6 months (Supplementary Fig. S2) (Regression analysis: 3 months, p = 0.047, 6 months, p = 0.020). Coral settlers on Type II tetrapods appeared therefore more vulnerable to mechanical damage as tetrapods dispersed across the reef.

The topography of the outplanting sites significantly affected the survival of *F. fragum* settlers as they were 8.7 and 5.2 times less likely to survive in areas with Low structural complexity compared to those seeded in Medium and Highly complex reefs after one year (Fig. 4c) (K-M: χ^2^_2_ = 13.8, p = 0.001, Supplementary Table S7). The five one-year-old individuals that were still alive in Low complexity areas had however grown to equal sizes as those in Medium and High complexity reefs (Welch’s F-test: F_2,20_ = 0.53, p = 0.59, Supplementary Table S8).

### SU yield

**Figure 5:**
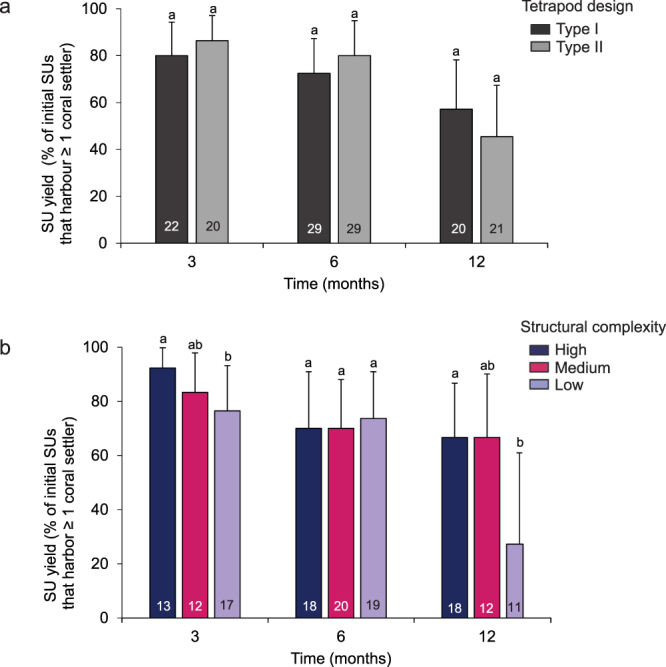
Seeding unit (SU) yield. Proportion of initial SUs that could be recovered through time and that still harboured at least one live *F. fragum* individual between (**a**) Type I and II tetrapods and (**b**) Low, Medium and High levels of structural complexity. Error bars are 95% confidence intervals as determined with Clopper-Pearson’s exact method. Letters above bars indicate significantly different groups as determined with Fisher’s exact test. Numbers within bars indicate sample sizes and are the number of outplant locations that were monitored. The latter increases between 3 and 6 months because not all outplant locations could be monitored at t = 3 months due to logistical constraints.

Overall, 56% of initial SUs still harboured at least one *F. fragum* individual after one year and SU yield was similar between both tetrapod designs (Fig. 5a) (Fisher’s exact test: 3 months, p = 0.69, 6 months, p = 1.00, 12 months, p = 1.00, Supplementary Table S9). The SU yield after one year was however 2.5 fold lower in habitats with Low structural complexity (27%, LCL = 6%, UCL = 60%) compared to Medium and High complexity reefs combined (67%, LCI = 47%, UCL = 83%) (Fig. 5b) (Fisher’s exact test: p = 0.046, Supplementary Table S10). The effectiveness of the seeding approach was therefore reduced in areas with low relief, and traditional outplanting techniques using binding materials likely represent a more effective strategy in such habitats (Table 3). However, except for low complexity areas, seeding SUs resulted in similar SU yields after one year (67%) compared to non-seeding restoration techniques using some form of binding materials (range: 25% to 70%, Table 3).

**Table 3:** Cost-effectiveness of different outplanting techniques.

Source	Coral species	Substrate design	Outplanting approach	Outplanting materials	Nursery phase	Reef structural complexity	Person-hour per hectare^a,b^	Cost per hectare^c^	Settler survival after one year (%)	SU yield after 1 year (%)	SU cost after 1 year^a^
Current study	*Favia fragum*	tetrapod	seeding	none	3 weeks	Low	48	6800	2.1	27	2.50
						Medium	48	6800	15.1	67	1.00
						High	48	6800	10.1	67	1.00
Chamberland *et al*.^19^	*Acropora palmata*	tripod	transplanting	epoxy	1 year	n.a.	1667	33400	n.a.	70	4.80
Chamberland *et al*.^9^	*Acropora palmata*	tripod	transplanting	cable-tie, rope, nails	2 weeks	n.a.	690	22200	12.7	27	8.30
Guest *et al*.^11^	*Acropora millepora*	plug-in	transplanting	drill, epoxy	7 months	n.a.	3200	45200	n.a.	25	17.90
					14 months	n.a.	3200	45200	n.a.	35	12.90
					19 months	n.a.	3200	45200	n.a.	43	10.50
Villanueva *et al*.^13^	*Acropora valida*	tox	transplanting	epoxy	6 months	n.a.	1086	25100	n.a.	n/a	n/a

^a^Assuming 10,000 Seeding Units (SUs) are needed to restore one hectare of reef. ^b^Assuming 10 persons are needed to restore one hectare of reef. ^c^Costs are in US dollars.

### Cost-effectiveness of seeding sexually propagated corals

Outplanting 10,000 SUs using binding materials requires 690 to 3,200 person-hours, whereas ‘seeding’ the same number of SUs in reef crevices could be achieved in 48 person-hours (Table 3). Because SUs could be outplanted rapidly (8.6 seconds per SU (±0.5SE, n = 59) and without purchasing binding materials, seeding 10,000 SUs cost $7,000 USD compared to $22,000–$45,000 USD if coral settlers were outplanted using other techniques (Table 3). When accounting for SU loss and settler mortality during the first year following the outplanting, remaining SUs each cost $1.00 USD (Medium and High complexity habitats) to $2.50 USD (Low complexity habitats), excluding expenses for larval rearing (Table 3).

## Discussion

Coral restoration can only be an effective management tool if it is cost-effective and can be applied at scales similar to the processes that cause their decline^1,4^. Current practices for restoring degraded reefs are generally expensive and labour intensive, making them unviable management options for restoration across larger spatial scales (i.e., >1 hectare). In this study we examined the possibility to improve the cost-effectiveness of outplanting sexually propagated corals by reducing the labour required to manually outplant them on the reef. We tested two tetrapod-shaped substrates for coral settlement, tetrapod Type I and II (Fig. 1), which were designed to be deployed without the need for attachment or binding materials and still become permanently attached at their outplant location. These tetrapods with coral settlers were outplanted by simply wedging them in crevices in the reef framework, which only took 1.5 to 7% of the time required to outplant sexually produced corals using traditional outplanting methods (Table 3). While tetrapods moved around (6 cm week^−1^) during the first two weeks after outplanting, they rapidly became stuck thereafter (Tables 1 and 2). One year after they were seeded onto the reef, 76% of tetrapods could still be recovered across all three levels of reef structural complexity, where they had become firmly lodged in crevices and/or cemented to the reef framework by encrusting benthic organisms (Fig. 3a–d, Table 2). Our findings therefore suggest that seeding SUs represents a relatively cheap and fast method to reintroduce corals to degraded reefs with long-term results similar to studies whereby SUs are manually secured to the benthos in habitats with medium to high structural complexity (Table 3).

### Effectiveness of seeding sexually propagated corals

Theoretically, only one remaining live and healthy coral colony per outplanted SU is required to eventually yield a successful restoration outcome^4^. The proportion of initial SUs harbouring at least one coral individual through time therefore serves as a measure to compare the effectiveness of different restoration techniques. In the current study, the SU yield in reefs with moderate to high topographic complexities was 1.5 fold higher than the median effectiveness of earlier outplanting efforts (45%, Table 3), but much less effective on reefs with low levels of structural complexity. In such areas, tetrapods dispersed easily (Table 1), increasing the probability that coral settlers became abraded or crushed, and often remained unattached until the end of the experiment (Table 2). Combined, this resulted in a 5 to 9-fold increase in settler mortality (Fig. 4c) and 2.4 times lower SU yield (Fig. 5b) relative to areas with higher levels of structural complexity. Thus, seeding the tetrapods may not be successful in areas exposed to high wave energy or with low structural complexity unless their design is improved to promote attachment in such areas. Securing the SUs with binding materials, such as epoxy, likely represents a more effective approach than seeding current tetrapod designs.

### Cost-effectiveness of seeding sexually propagated corals

Overall, the new tetrapod-shaped substrates could be outplanted efficiently with low costs for labour and materials, enabling 10,000 SUs to be seeded in one hectare of reef within 48 h at a cost of $7,000 USD (Table 3). This represented a 5 to 18 fold reduction in costs of the actual outplanting process compared to traditional outplanting techniques. The production of the tetrapods themselves accounted for a large fraction of the production cost for a single one-year-old SU ($0.50 USD) (Supplementary Table S2), indicating that the cost-effectiveness of this new technique could be further improved if tetrapods would be produced industrially or at lower costs. Because the outplanting phase normally incurs a large proportion of the costs associated with coral restoration activities (~30%)^4^, the ‘seeding’ of SUs, if combined with other economical but effective larval rearing techniques, could significantly reduce the costs of restoring degraded reef systems. Under such scenario, costs of reef restoration would become more comparable to the costs of existing mangrove and salt marsh restoration programs (<$10,000 USD per hectare)^22^, allowing the application of coral restoration across much larger scales.

### Optimization of the tetrapod designs

While the tested tetrapod designs reduced the amount of labour and costs during the outplanting phase, they were not optimal for coral settler survival and growth. For example, the average survival of *F. fragum* settlers was only 9.6% after one year (see: Ritson-Williams *et al*.^20^, Vermeij^37^, and Hartmann *et al*.^38^ for an overview of factors contributing to settler mortality in Curaçao), and very low compared to the 42% survival reported for *F. fragum* settlers settled on CCA chips in Belize^30^. While the tetrapods were successfully colonized by thin CCA communities that facilitate larval settlement and metamorphosis, their light-exposed upper surfaces became rapidly overgrown by algal turfs once outplanted on the reef (Supplementary Fig. S3), which likely contributed to the high mortality rates of *F. fragum* settlers during the first three months following the outplant^39^. Because algal propagules and spores easily adhere to the porous texture of concrete structures^40^, producing the tetrapods (including microstructures such as grooves) from non-porous materials such as glass or glazed ceramics, rather than from concrete, could prevent the formation of turf algal communities on the tetrapods, and subsequently enhance the survival and growth of settled corals.

*Favia fragum* larvae preferentially settled inside the tetrapods’ grooves where they experienced lower post-settlement mortality rates. Grooves provide spatial refuges from incidental grazing of newly settled corals by herbivorous fishes and urchins^27,28^, and should therefore always be considered in settlement substrate designs to enhance settler survival^8^. Grooved surfaces accounted for less than a third of the current tetrapods’ total surface area (Supplementary Table S1), and future designs could likely be improved by increasing the amount of these microhabitats.

Because coral individuals that remain as single polyps past the age of one year often no longer enter the two or more polyp stage, the survival of one-polyp settlers per se is not indicative of effective recruitment^14^. Here, 62% of one-year-old *F. fragum* individuals formed two- to seven-polyp colonies (Supplementary Fig. S3), and most small-sized and one-polyp settlers were found on the cryptic undersides of the tetrapods (Fig. S3), where growth is repressed by low light availability^41,42^. Corals that settle on the undersides of artificial settlement substrates should be able to rapidly grow into light-exposed areas, where they will benefit from higher light levels^41^. Sub-cryptic surfaces (e.g., vertical walls, horizontal holes or crevices on the upward facing parts of settlement substrates), rather than fully cryptic surfaces such as the undersides of the tested tetrapods, would likely represent better microhabitats to be included in future tetrapod designs to allow a certain degree of protection to new settlers, without compromising their chances to grow into light-exposed areas.

## Conclusions

Sexually propagating corals to restore depauperate coral populations has thus far been a time consuming, technically challenging and an expensive undertaking^4^, and as a result has only been applied on small scales (≤2,000 SUs per restoration site). By avoiding the need for outplanting corals using binding materials, the seeding approach allows the deployment of large numbers of young corals in a very short amount of time and at low cost. This technique was most effective in reefs with moderate to high topographic complexity, where tetrapods rapidly became stabilized within the reef framework and resulted in a high SU yield relative to traditional outplanting methods. While we acknowledge that improvements can still be made in future tetrapod designs to optimize the survival and growth of coral settlers, this novel approach nonetheless represents a next step towards large-scale restoration using sexually propagated corals.

## Electronic supplementary material

Supplementary figures

Supplementary tables

Supplementary dataset
