# Supplementary material for: New Seeding Approach Reduces Costs and Time to Outplant Sexually Propagated Corals for Reef Restoration
Source: Sci Rep. 2017 Dec 22;7:18076. doi: 10.1038/s41598-017-17555-z (PMC5741773; doi:10.1038/s41598-017-17555-z)
Supplement: Supplementary file 1 — Supplementary figures [file 41598_2017_17555_MOESM1_ESM.pdf]

New Seeding Approach Reduces Costs and Time to Outplant Sexually Propagated Corals for Reef Restoration

Valérie F Chamberland[1,2,3*¶], Dirk Petersen[1¶], James R Guest[1,4], Udo Petersen[5], Mike Brittsan[1,6], Mark JA Vermeij[2,3]

Supplementary figures

[1] SECORE International, Hilliard, OH, USA

[2] CARMABI Foundation, Willemstad, Curaçao

[3] Freshwater and Marine Ecology, Institute for Biodiversity and Ecosystem Dynamics, University of Amsterdam, Amsterdam, The Netherlands

[4] School of Natural and Environmental Sciences, Newcastle University, Newcastle upon Tyne, United Kingdom

[5] Faculty of Mechanical Engineering, Kempten University of Applied Sciences, Kempten, Germany

[6] Columbus Zoo and Aquarium, Powell, OH, USA

* Corresponding author

E-mail: v.chamberland@secore.org (VFC)

[¶] These authors contributed equally to this work.

Figure S1

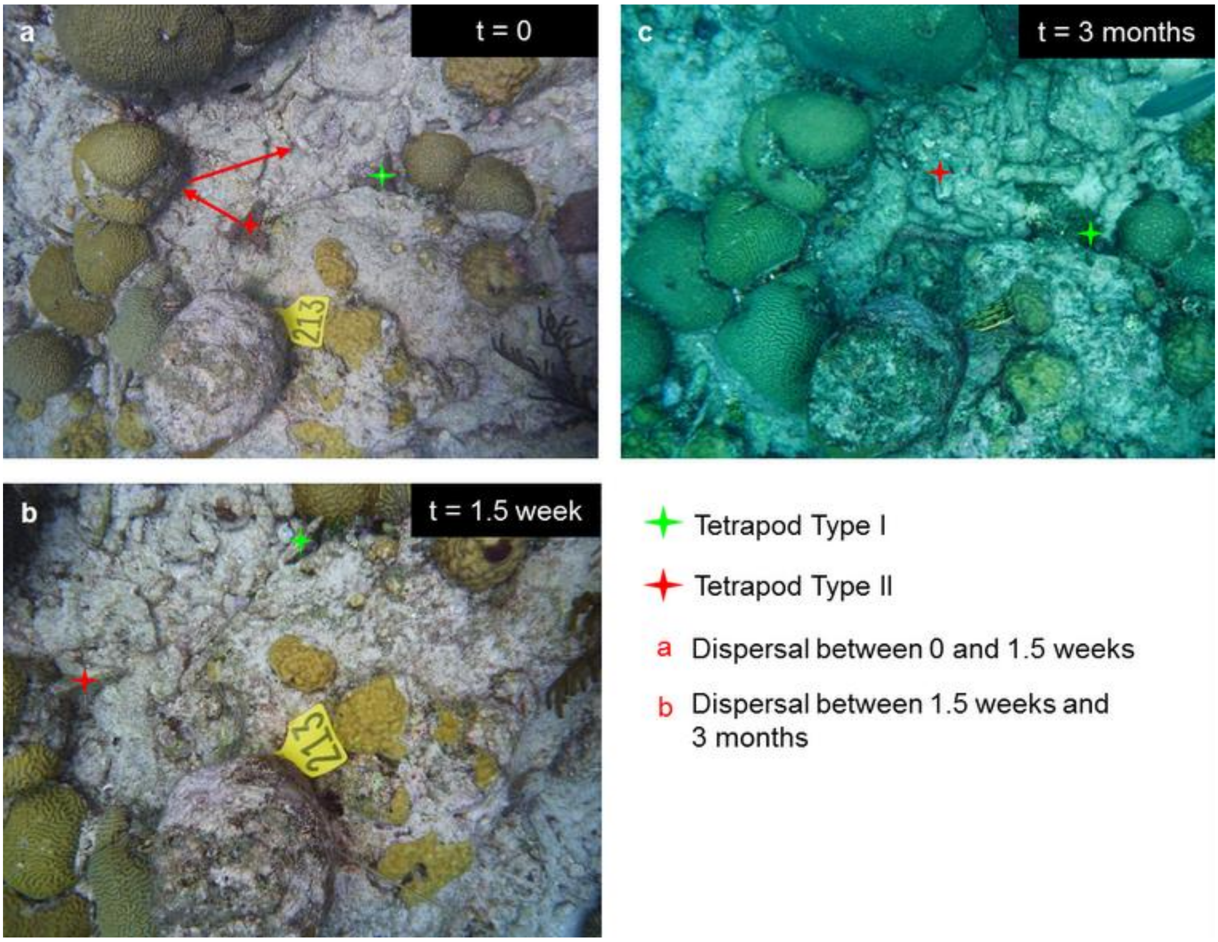

Figure S1. Example of photo-analysis of tetrapod dispersal for a period of three months. Type I (green markers) and Type II (red markers) tetrapods were located in overview pictures of each outplant location taken after (**a**) t = 0, (**b**) t = 1.5 week, and (**c**) t = 3 months. The tags and natural landmarks served as references to help compare the position of each tetrapod through time, while the length of the tags served as a scale to measure the distance between the previous and current position (indicated by arrows of corresponding colours) of the tetrapods ImageJ. In this example, tetrapod Type I stayed in place during the first three months of the experiment, while tetrapod Type II dispersed for a total distance of *a* + *b.* Photos by VFC.

Figure S2

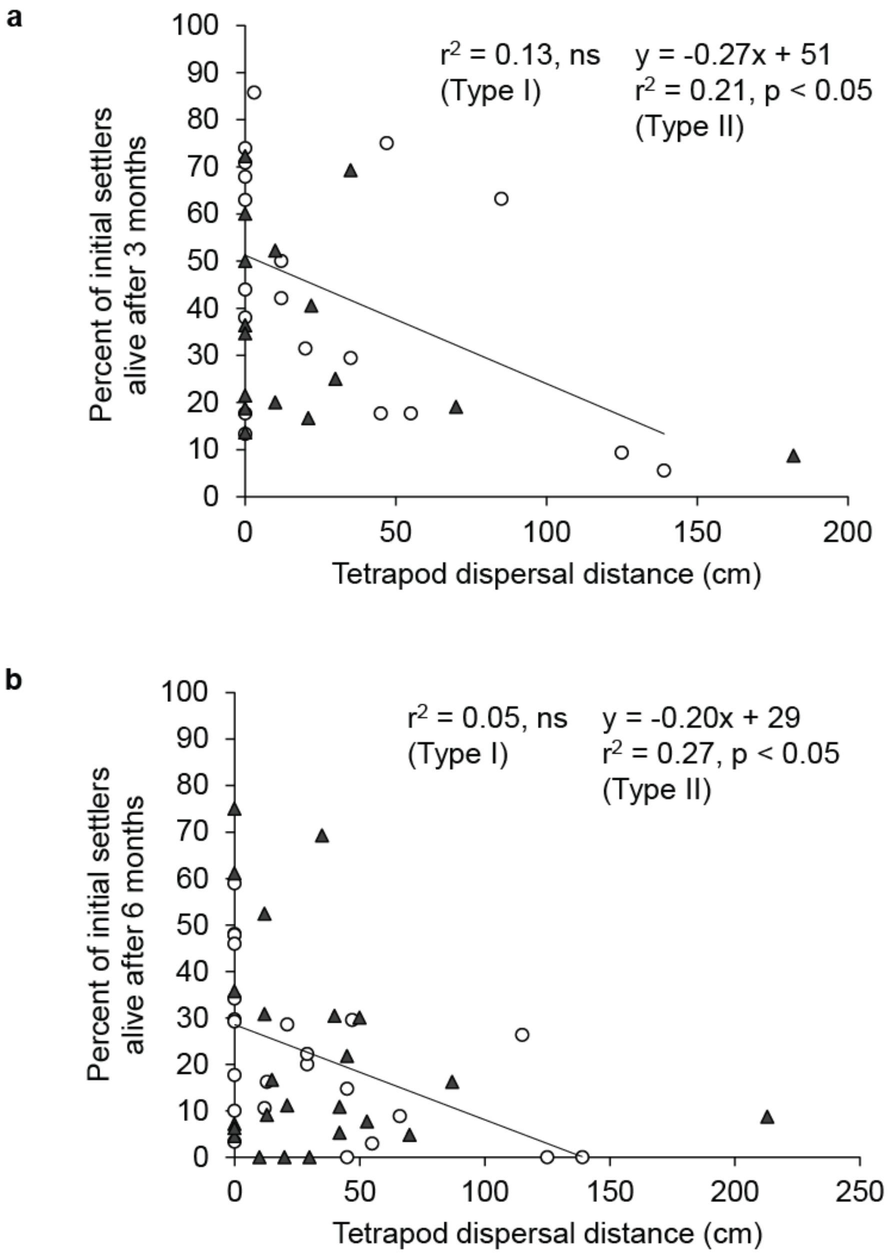

Figure S2. Effect of tetrapod dispersal on the survival of coral settlers.

Regression analysis with dispersal distance as predictor variable and with *Favia fragum* settler survival as response variable after (**a**) 3 and (**b**) 6 months. Dark triangles are tetrapod Type I and clear circles are Type II.

Figure S3

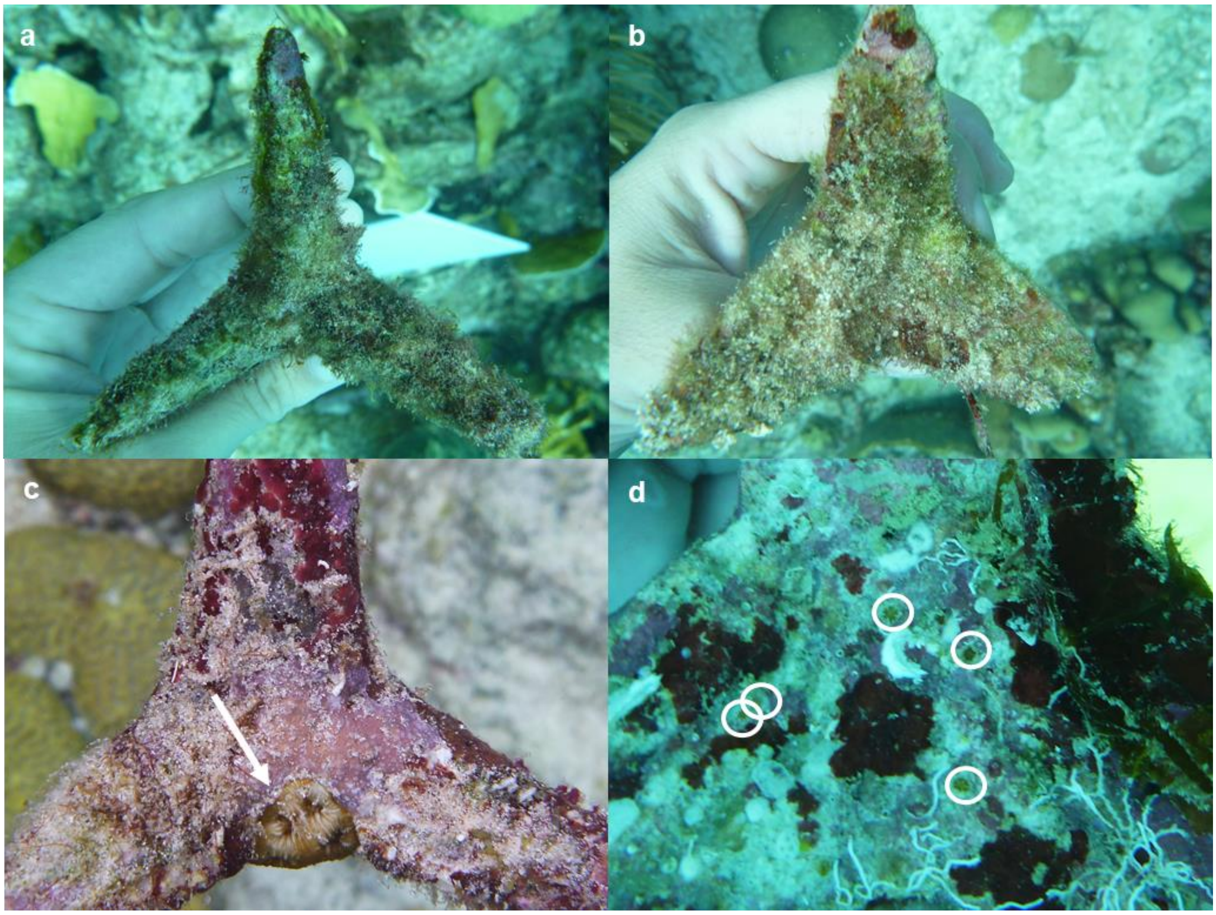

Figure S3. Examples of benthic communities and of coral settlers that grew on the tetrapods. Three months after they were deployed on the reef, the tetrapod's light-exposed surfaces on both (**a**) Type I and (**b**) Type II designs had become overgrown by communities of turf algae. (**c**) *Favia fragum* settlers that survived on the light-exposed surfaces of the tetrapods typically formed multi-polyp colonies (shown by white arrow) and reached larger sizes than those located on the (**d**) cryptic undersides of the tetrapods. The latter often still consisted of small, single-polyp individuals (shown by white circles) after one year. Photos by VFC.
